# Prevalence and correlates for ADHD and relation with social and academic functioning among children and adolescents with HIV/AIDS in Uganda

**DOI:** 10.1186/s12888-017-1488-7

**Published:** 2017-09-22

**Authors:** Richard Stephen Mpango, Eugene Kinyanda, Godfrey Zari Rukundo, Jonathan Levin, Kenneth D. Gadow, Vikram Patel

**Affiliations:** 1 Mental Health Project, MRC/UVRI Uganda Research Unit on AIDS, P. O. Box 49, Entebbe, Uganda; 2 Department of Psychiatry, Mbarara University of Science and Technology, P. O. Box 1410, Mabarara, Uganda; 3 Statistical Section, MRC/UVRI Uganda Research Unit on AIDS, Entebbe, Uganda; 4 School of Public Health, Faculty of Health Sciences, University of Witwatersrand, Johannesburg, South Africa; 5 Department of Psychiatry, Health Sciences Centre, Stony Brook University, Stony Brook, NY 11794-8790 USA; 6 Department of Global Health and Social Medicine, Harvard Medical School, Massachusetts, USA; 7 Department of Psychiatry, Makerere College of Health Sciences, Kampala, Uganda

**Keywords:** ADHD, Children/adolescents, HIV, Prevalence, Correlates

## Abstract

**Background:**

Aim of this study was to determine the prevalence of attention-deficit/hyperactivity disorder (ADHD), its associated correlates and relations with clinical and behavioural problems among children and adolescents with HIV/AIDS (CA-HIV) attending five HIV clinics in central and South Western Uganda.

**Methods:**

This study used a quantitative design that involved a random sample of 1339 children and adolescents with HIV and their caregivers. The Participants completed an extensive battery of measures including a standardized *DSM-5* referenced rating scale, the parent version (5–18 years) of the Child and Adolescent Symptom Inventory-5 (CASI-5). Using logistic regression, we estimated the prevalence of ADHD and presentations, correlates and its impact on negative clinical and behavioural factors.

**Results:**

The overall prevalence of ADHD was 6% (*n* = 81; 95%CI, 4.8–7.5%). The predominantly inattentive presentation was the most common (3.7%) whereas the combined presentation was the least prevalent (0.7%). Several correlates were associated with ADHD: socio-demographic (age, sex and socio-economic status); caregiver (caregiver psychological distress and marginally, caregiver educational attainment); child’s psychosocial environment (quality of child-caregiver relationship, history of physical abuse and marginally, orphanhood); and HIV illness parameters (marginally, CD4 counts). ADHD was associated with poor academic performance, school disciplinary problems and early onset of sexual intercourse.

**Conclusions:**

ADHD impacts the lives of many CA-HIV and is associated with poorer academic performance and earlier onset of sexual intercourse. There is an urgent need to integrate the delivery of mental health services into routine clinical care for CA-HIV in Sub-Saharan Africa.

**Electronic supplementary material:**

The online version of this article (10.1186/s12888-017-1488-7) contains supplementary material, which is available to authorized users.

## Background

The Joint United Nations Programme on AIDS estimates that 88% of the global 3.3 million children and adolescents living with HIV (CA-HIV) reside in Sub-Saharan Africa [1]. Most of these CA-HIV who were perinatally infected with HIV are at risk of developing emotional and behavioural problems [2]. Although many countries in Sub-Saharan Africa including Uganda [3] have adopted the WHO policy recommendation [4] that calls for the integration of mental health services into HIV care, most HIV clinics on the continent have yet to implement them [5].

In Uganda, prevalence of HIV in the general population has stabilised around 7.4% [6], with highest rates in the Central Region (10.6%) and the lowest in Mid-Eastern Region (4.1%). Prevalence is higher among women (8.3%) than men (6.1%); 3.7% of young women and men between 15 and 24 years of age are HIV-positive [3]. There is fairly compelling evidence that HIV contributes to neurocognitive impairment and possibly neuropsychiatric illnesses [7–10]. CA-HIV experience relatively high rates of psychopathology [11–15] that could arise from the direct and indirect effects of HIV including the psychosocial problems of family disruptions; poor social support; and a high burden of negative life events, stigma, poverty and chronic ill health [16] and the burden of long-term treatment with antiretroviral therapy (ART) [17].

Previous studies have found that ADHD is one of the most common psychiatric disorders among CA-HIV (Scharko, as cited in Mellins and Malee, 2013) [2]. However, little is known about ADHD among CA-HIV in Sub-Saharan Africa. Most of what is known comes from two studies [15, 18] conducted in Kenya and South Africa. The Kenyan study reported a prevalence of 12.2% as based on both parent and youth report [18], whereas the South African study found rates of 88% and 17% based on parent and teacher ratings, respectively [15]. The rates of ADHD reported in the Kenyan and South African studies differed significantly possibly due to differences in the assessment instruments used. Whereas the Kenyan study used the MINI International Neuropsychiatric Interview for children and adolescents (MINI-KID) [18], a *DSM IV* based psychiatric assessment tool that undertakes both a symptom count and assessment of functional impairment, the South African study used the Swanson, Nolan and Pelham rating scale [15], a *DSM IV-*based psychiatric assessment tool that only assesses symptom count. To date, no study has established the prevalence of ADHD among CA-HIV in Uganda.

Although it is widely recognized that ADHD is highly heritable [19], a wide range of variables are associated with symptom severity in CA-HIV [2], and these include impaired social and academic functioning [2, 17, 20–24], which can negatively affect quality of life. However, relatively little is known about child, family, and illness characteristics associated with severity of ADHD among CA-HIV in East Africa or their implications for functional outcomes. The primary objectives of the present study were to (a) document the prevalence of ADHD among a large sample of CA-HIV attending rural and urban HIV clinics in Uganda, (b) characterize the relation of ADHD severity with a wide range of commonly studied clinical correlates, and (c) examine the relation of ADHD with important indices of academic and social functioning. We were particularly interested in ADHD because it is associated with emotional and behavioural dysregulation thus increasing the risk of impaired decision making, poor impulse control, risky sexual behaviour, and aggression among CA-HIV [2].

## Methods

### Participants

The study comprised a random sample of 1339 child/adolescent-caregiver dyads from the five HIV clinics in rural (Masaka) and urban (Kampala) Uganda. To be eligible for the study, CA-HIV had to be between 5 and 11 and 12–17 years of age, respectively, and caregivers had to be at least 17 years of age. Both CA-HIV and caregivers had to speak English or Luganda (the local language spoken in the study areas) and plan to reside in the study area for the subsequent 12 months. Exclusion criteria were concurrent enrollment in another study (which applied to only one site), need of immediate medical attention, and unable to understand the study’s assessment instruments.

### Measures

The assessment battery comprised standardised, locally translated psychosocial instruments [16, 17, 25–35]. Study variables reported in this paper are described in Additional file 1: Table S1 arranged according to the conceptual framework. ADHD was established using a clinically practical, *DSM-5*-referenced, behaviour rating scale, the Child and Adolescent Symptom Inventory-5 (CASI-5) [34], which was previously used to study ADHD in CA-HIV in the United States [35, 36]. CASI-5 items can be scored in several different ways, and in the present study we used the symptom count cut-off score. Symptom count cut-off score indicates whether child/adolescent has the prerequisite number of symptoms necessary for a *DSM-5* diagnosis. The CASI-5 also provides an algorithm that we used to generate the different ADHD presentations.

### Procedures

To obtain the required sample of 268 study participants per study site, research assistants identified potential participants from the patient register. Those who met eligibility criteria were then invited to enroll into the study. This procedure was repeated each clinic day until the required sample size of each site was attained. About 2% of eligible participants were not included owing to a number of reasons including participation in another ongoing study, inability to contact the caregiver to obtain consent, or refusal to give consent by the caregiver.

The study protocol was administered by trained psychiatric nurses and psychiatric clinical officers supervised by a clinical psychologist (RM) and overseen by a psychiatrist (EK). Interviews were conducted in two parts, each part lasting 30 min with a tea break in between. Psychosocial assessments used for the first time in Ugandan, which included the CASI-5 [34], underwent a translation and local adaptation process described in a separate paper [37]. Briefly, this entailed separate forward and back translations by teams of mental health professionals and lay people conversant in both English and Luganda; a consensus workshop of translation teams to derive the versions with the most acceptable face validity; and administration to CA-HIV/caregiver dyads to refine the terminology in order to enhance clarity.

### Statistical analyses

**Fig. 1:**
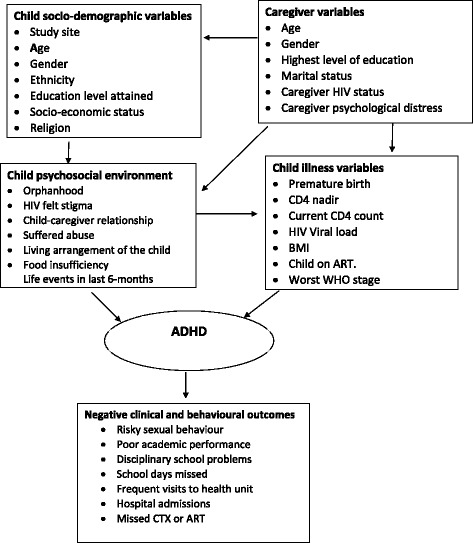
Conceptual framework for clinical correlates of ADHD

Prevalence of the three ADHD presentations (predominantly inattentive, predominantly hyperactive-impulsive, and combined) as well as any ADHD was estimated with 95% confidence intervals by age-group and gender. Clinical correlates for ADHD were determined by fitting multiple logistic regression models, using the approach recommended by Victora et al. [38]. These variables were divided into four groups: socio-demographic, caregiver, child’s psychosocial environment, and CA-HIV health status (see Fig. 1). First, an initial prediction model was selected based only on socio-demographic variables; study site (as a design variable), sex and age were included in all models as a priori confounders, while other socio-demographic variables were excluded from the model if they were not significant at the 10% level. Caregiver characteristics were then added to the socio-demographic model that had been selected, and those variables that were not statistically significant at the 10% level were removed. Next, psychosocial environment variables were then added to the socio-demographic and caregiver model, and variables that were not statistically significant at the 10% level were removed. Analyses were conducted separately for children and adolescents as some variables were obtained for adolescents but not on children. Then, variables related to the health status of CA-HIV were added to the previous model, and those that were not significant at the 10% level were removed. Finally, any of the initial variables that were no longer significant at the 15% level were omitted. This liberal *p*-value for potential confounders is advocated by some experts, e.g., Royston et al. [1].

We then examined the association of ADHD with several functional outcomes: poor academic performance at school, poor social functioning at school (experienced problems at school), number of visits to the health unit in the past month, hospital admissions in the last month, non-adherence to HIV treatment and risky sexual behaviour (onset of sexual intercourse) (see Additional file 1 for a detailed description of these variables). For each of these outcomes, the association with ADHD was assessed by fitting a logistic regression model, adjusting for study site (as a design variable), sex, age and educational level. For visit to a health unit and hospital admission, adjustment was also made for current CD4 count, whereas for adherence, adjustment was made for whether or not the CA-HIV was receiving ART (which determined how non-adherence was measured). Associations were assessed separately for each ADHD presentation.

To adjust for multiple testing we followed the suggestion of Bender and Lange [37] and regarded the study as an exploratory study in which significance tests were used for descriptive purposes rather than for decision making. Thus, findings should be viewed as preliminary, and further work is required to carry out confirmatory studies. This approach is supported by Vittinghoff et al. [39] who noted that in an exploratory analysis, it is not clear how many comparisons should be corrected for, but stressed the need for cautious interpretation of any findings.

### Ethical considerations

The study obtained ethical approvals from the Uganda Virus Research Institute’s Research and Ethics Committee, the Ethics Committee of the London School of Hygiene and Tropical Medicine and the Uganda National Council of Science and Technology. Participants voluntarily provided consent (caregivers)/assent (CA-HIV). Participants found to have a psychiatric disorder were provided with psycho-education and referred to their local mental health departments.

## Result

### Characteristics of study participants

Briefly, 64% of CA-HIV were between 5 and 11 years, and 36% were between 12 and 17 years (see Additional file 2 for a detailed description of the study sample). The urban and rural study sites contributed equally. There slightly were more females (52%) than males (48%). With regard to HIV illness parameters, 88% had CD4 counts equal or greater than 350 cells/μL, and most (95%) of the participants were receiving ART.

### Prevalence of ADHD

Six percent (6.0%; 95% CI, 4.8–7.5%) of CA-HIV met CASI-5 symptom count criteria for at least one ADHD presentation (Table 1). For specific presentations, rates were 3.7% for the predominantly inattentive (I) presentation, 1.6% for the predominantly hyperactive-impulsive (HI) presentation, and 0.7% for the combined (C) presentation. Among children the most frequent ADHD presentation was ADHD-I (3.0%), followed by ADHD-HI (2.0%) with ADHD-C (0.6%) being the least frequent. Among adolescents ADHD-I (4.8%) was also the most frequent, but both ADHD-HI (1.0%) and ADHD-C (1.0%) were second. The rank order of presentations was the same for both males and females.

**Table 1:** The prevalence of ADHD based on CASI-5 Symptom Count Cut-off scores

		ADHD-I	ADHD-HI	ADHD-C	Any ADHD
Subgroup	*N*	*n* (%)	*n* (%)	*n* (%)	*n* (%)
Symptom Count Cut-off	1339	49(3.7%)	22 (1.6%)	10 (0.7%)	81 (6.0%)
Children	860	26 (3.0%)	17 (2.0%)	5 (0.6%)	48 (5.6%)
Adolescents	479	23 (4.8%)	5 (1.0%)	5 (1.0%)	33(6.9%)
Male	638	30 (4.7%)	13 (2.0%)	6 (0.9%)	49(7.7%)
Female	699	19 (2.7%)	9 (1.3%)	4 (0.6%)	32(4.6%)

NOTE: *ADHD*attention-deficit/hyperactivity disorder, *I* inattentive presentation, *HI* hyperactive-impulsive presentation, *C* combined presentation, *CASI* Child and Adolescent Symptom Inventory

#### Clinical correlates of ADHD inattentive

Variables associated with ADHD-I symptom count cut-off score were increasing age (aOR of 1.14 per 1 year increase in age, *p* = 0.004), socio-economic status (aOR of 1.91 per unit increase in SES, *p* = 0.009), caregiver psychological distress scores (aOR of 1.11 per unit increase in psychological distress scores of caregiver, *p* = 0.004), and quality of child-caregiver relationship (aOR of 1.34 per unit deterioration in quality of the child-caregiver relationship scores, *p* < 0.0001) (see Additional file 3). Higher caregiver educational status (*p* = 0.08) and CD4 counts (*p* = 0.06) approached significance.

#### Clinical correlates of ADHD hyperactive-impulsive

Variables associated with ADHD-HI symptom scores were increasing caregiver psychological distress scores (aOR of 1.11 per unit increase in psychological distress scores of caregiver, *p* = 0.02), quality of child-caregiver relationship (aOR of 1.20 per unit deterioration in quality of the child-caregiver relationship scores, *p* = 0.09), and only assessed among adolescents, having ever been beaten (aOR = 5.0, *p* = 0.02) (see Additional file 3).

#### Clinical correlates of ADHD combined

Variables associated with ADHD-C symptom scores were caregiver psychological distress (aOR of 1.16 per unit increase in psychological distress scores of caregiver, *p* = 0.04) (see Additional file 3). Orphanhood was marginally significant (*p =* 0.05), with rates highest for those CA-HIV with no living parent compared to those who had at least one living parent (aOR = 11.03, *p* = 0.05).

#### Clinical correlates of any ADHD

Variables associated with having any ADHD presentation were increasing socio-economic status (aOR of 1.84 per unit increase in SES, *p* = 0.004), caregiver psychological distress scores (aOR of 1.11 per unit increase in psychological distress scores of caregiver, *p* = 0.001), quality of child-caregiver relationship (aOR of 1.32 per unit deterioration in quality of the child-caregiver relationship score, *p* < 0.0001) (see Additional file 3). Caregiver educational level was marginally significant (*p* = 0.06).

### Association between ADHD presentation and outcome

ADHD-I presentation and having any ADHD were associated with poor academic performance (see Table 2), and having any ADHD was associated with having problems at school. ADHD-I and having any ADHD were marginally associated with early initiation of sexual intercourse (*p* = 0.07). None of the ADHD presentations were associated with non-adherence to HIV treatment, number of hospital visits, or number of hospital admissions.

**Table 2:** Association between ADHD and adverse outcomes

Outcome	ADHD-I aOR;95% CI; *P*-value	ADHD-HI aOR;95% CI; *P*-value	Any ADHD aOR;95% CI; *P*-value
Onset of sexual intercourse	3.43 (0.91; 12.92) *P* = 0.07	0.87 (0.07; 10.59) *P* = 0.92	2.93 (0.86; 9.97) *P* = 0.09
Poor academic performance	3.72 (2.02; 6.85) *P* < 0.001	1.43 (0.53; 3.82) *P* = 0.48	3.85 (2.26; 6.55) *P* < 0.001
Any problems at school	1.53 (0.79; 2.94) *P* = 0.21	2.11 (0.88; 5.02) *P* = 0.09	2.30 (1.35; 3.92) *P* = 0.002
Any visit to the health Unit	0.81 (0.30; 2.18) *P* = 0.68	0.66 (0.15; 2.99) *P* = 0.59	0.70 (0.29; 1.68) *P* = 0.43
Hospital Admission	Too few cases to model	2.31 (0.29; 18.13) *P* = 0.43	0.52 (0.07; 3.91) *P* = 0.52
Non-adherence to HIV treatment	1.58 (0.47; 5.28) *P* = 0.46	0.56 (0.06; 5.47) *P* = 0.62	1.16 (0.37; 3.59) *P* = 0.80

NOTE: *ADHD* attention-deficit/hyperactivity disorder, *I* inattentive presentation, *HI* hyperactive-impulsive presentation, *C* combined presentation, *CASI* Child and Adolescent Symptom Inventory

## Discussion

This study aimed to determine the prevalence of ADHD among CA-HIV in Uganda and associations of ADHD with commonly studied mental health clinical correlates and social, academic and clinical functioning. The prevalence of caregiver-rated ADHD was 6%, which is similar to caregiver-reported CASI ADHD rates for non-HIV, community-based samples in the United States [40, 41]. When we adopted a more clinically oriented criterion for any ADHD based on both symptom count cut-off score and impairment cut-off score, the prevalence of any ADHD was 2.2%, which underscores the importance of considering criteria for defining ADHD when comparing results across studies. With regard to CA-HIV, a study conducted in the United States that used CASI [17, 35] symptom count cut-off scores found that 16% met criteria for ADHD according to caregiver ratings [36]. These findings seem to suggest that the rate of ADHD in the United States study is much higher than that in Uganda, at least among HIV populations. However, Gadow et al. [17] also found the prevalence of ADHD among uninfected youth in the United States from HIV-affected families was similar to CA-HIV (12.6%), and rates for both groups (infected and affected) were higher than comparable rates for population-based samples [40, 41]. The authors noted that one plausible explanation for these differences between infected/affected youth and population-based samples in the United States is the much higher percentage of minority youth among the former and the well-established relation of minority status with mental health concerns, clinical correlates, and less than optimal health care. To this we would add that socio-cultural differences in symptom grading among caregivers in more industrialised Western countries as compared to the more agrarian Ugandan environment (where the caregivers are expected to have a tolerance for psychiatric symptomatology as compared to their Western counterparts) may also contribute to disparate rates of ADHD.

In Uganda the most common ADHD presentation was ADHD-I, followed by ADHD-HI with ADHD-C being the least prevalent. This relative distribution of ADHD presentations was similar for children and adolescents with the exception that rates of ADHD-HI and ADHD-C were identical among adolescents. In the United States study conducted by Gadow and colleagues [17], among their younger cohort (6–12 years old) both ADHD-I and ADHD-HI were tied in first place. Among their older cohort (13–18 years old), ADHD-I was more common than ADHD-HI and ADHD-C, rates for which were identical. Conversely, the South African study by Zeegers and colleagues [15] found that caregiver ratings indicated ADHD-HI and ADHD-C were the most and least common, respectively, but teacher assessments of the same sample indicated ADHD-C was the most frequent presentation with ADHD-I and ADHD-HI both coming in second.

In our Uganda study, several clinical correlates were associated with the different ADHD presentations. Increasing age was associated with increased risk for ADHD-I, which is consistent with the findings from review of literature from nine studies conducted in Africa [42] and is likely explained by the increasing demands school curricula as youth grow older. We observed an increase in the risk of ADHD-I with increasing socio-economic status whereas previous studies of non-HIV samples conducted in the West have reported just the opposite [35, 37]. A possible explanation for our Uganda finding is that more affluent families are better able to pay school fees, and the school is arguably the primary setting in which ADHD-I symptoms are most problematic. In addition, higher socio-economic status is associated with caregiver educational attainment and greater sensitivity to their child’s academic success. Consistent with this interpretation, there was a marginally significant association between increasing caregiver educational status and rates of ADHD-I.

Increasing caregiver psychological distress was associated with higher rates of all ADHD presentations. Similar results have previously been reported by others both among an HIV [2] and a non-HIV [21] population in the West. Possible explanations suggested by others include heritability of ADHD (or any other mental health problems), stressful family and social environments associated with ADHD (or any other mental health problems), and the erosion of parenting capacities which often accompany a parent with HIV or mental illness [2, 21, 43]. In support of the latter, we found that deteriorating quality of the child-caregiver relationship (communication style and emotional reaction between the child and caregiver) was associated with all three ADHD presentations, which is consistent with the findings of others for both HIV [43] and non-HIV [44] samples. Suffering physical abuse (i.e., having ever been beaten) was associated with a fivefold increased risk of ADHD: HI in our adolescent-age sample, which is in accord with the extant literature [44]. It has been suggested that ADHD symptoms may illicit feelings of hostility in the caregiver causing them to use aversive behaviour management strategies with CA-HIV [44].

Additional variables associated with ADHD were orphanhood and CD4 counts. Orphanhood, which not surprisingly was highest among those who reported loss of both parents, was marginally associated with ADHD-C. Previous studies among HIV populations have reported the association between orphanhood and youths’ mental health [45, 46]. Decreasing CD4 counts were marginally associated with increase in the odds of ADHD-I, and prior studies undertaken in the United States report similar findings [2, 10, 35, 36, 43, 47, 48].

ADHD can have a substantial impact on school functioning and is associated with poor exam performance, grade retention, and failure to graduate from secondary school [49]. Cognitive deficits that may interfere with academic performance have commonly been reported among CA-HIV [22, 35, 50]. In our Ugandan sample, we found that any ADHD presentation and ADHD-I specifically were associated with poor academic performance. In addition, having any ADHD presentation was associated with problems at school, which is in accord with prior research involving CA-HIV [2, 35].

Early onset of sexual intercourse (only assessed among adolescents) was marginally associated (*p* = 0.07) with a two- to threefold increase of having any ADHD or ADHD-I (aORs of 2.93 and 3.43, respectively). Studies conducted elsewhere have reported a positive association between ADHD and risky sexual behaviour [21, 23, 35], which may have important implications for disease transmission. Although others have reported that among CA-HIV, co-occurring ADHD is associated with poorer adherence to HIV treatment, number of hospital visits, and hospital admissions [43, 50], this was not the case in our study.

This study has several strengths to include an exceptionally large sample of CA-HIV, comprehensive assessment battery, and cross-cultural orientation, there are also limitations. Because our analyses are cross-sectional, we cannot comment on the casual directions, but these will be addressed in future publications about the longitudinal component of the CHAKA study. Since ADHD symptoms are influenced by environmental variables and therefore different informants can disagree about symptom severity [51], our prevalence rates for ADHD in Uganda should be considered conservative estimates as we did not obtain CASI-5 ratings from our sample’s school teachers. Because we do not have a comparable sample of seronegative youth from the same geographic areas and environment, it is not possible to know whether relations between ADHD and putative mental health factors and functional outcomes are influenced by HIV status. This does not, however, detract from the clinical implications of our findings for CA-HIV. By design, the present study focused on CA-HIV living in Uganda, and owing to considerable cultural variation in East Africa, our results may not generalize to other countries in the region.

## Conclusions

In summary, approximately 6% of CA-HIV living in Uganda met *DSM-5* symptom count criteria for ADHD. Clinical ccorrelates of ADHD were reported for all domains (general socio-demographic, caregiver, psychosocial environmental and HIV illness). ADHD among CA-HIV was associated with poorer academic performance, school disciplinary problems, and earlier age of onset of sexual intercourse, all of which may have important implications for clinical management, quality of life, long-term outcome and possible disease transmission. Moving forward, there is a definite need to integrate mental health services into routine HIV care to include the development of cost-effective assessment and treatment strategies that have high probability of success in challenging intervention settings.

## Additional files

Additional file 1: Table S1. Data collection tools for the study. (DOCX 30 kb)

Additional file 2: Table S2. Characteristics of study participants. (DOC 47 kb)

Additional file 3: Table S3. Clinical correlates of ADHD. (DOCX 14 kb)

## Abbreviations

ADD: Attention Deficit Disorder
ADHD: Attention Deficit Hyperactive Disorder
AIDS: Acquired Immune Deficiency Syndrome
APA: American Psychiatric Association
ART: Anti-Retroviral Therapy
CA-HIV: Children and Adolescents infected with HIV/AIDS
CASI-4R: Child Adolescent Symptom inventory-4Revised
CBCL: Child Behavioural Checklist
CD: Conduct Disorder
CDC: Centre for Disease Control and prevention
CDI: Children’s Depression Inventory
CI-4R: Child Inventory-4Revised
CPRS: Conners’ Parent Rating Scale
CTX: Cotrimoxazole
DISC-IV: Diagnostic Interview Schedule for Children Version IV
HIV: Human Immunodeficiency Virus
IMPAACT/1055: International Maternal Paediatric Adolescent AIDS Clinical Trials Group 1055 study
KABC: Kaufman Assessment Battery for Children
KABC-II: Kaufman Assessment Battery for Children, second edition
ODD: Oppositional Defiant Disorder
PD: Psychiatric Disorder
YI-4R: Youth Inventory-4Revised
