# Supplementary material for: Prevalence and correlates for ADHD and relation with social and academic functioning among children and adolescents with HIV/AIDS in Uganda
Source: BMC Psychiatry. 2017 Sep 22;17:336. doi: 10.1186/s12888-017-1488-7 (PMC5610431; doi:10.1186/s12888-017-1488-7)
Supplement: Supplementary file 1 — Data collection tools for the study. (DOCX 30 kb) [file 12888_2017_1488_MOESM1_ESM.docx]

**Additional file 1: Description of Data Collection Measures**

|  | **Instrument used** | | **Description** | **Questions or categories (examples)** | **Remarks** | **Reference** |
| --- | --- | --- | --- | --- | --- | --- |
| **Socio-**  **demographic**  **factors** | Structured socio-demographic questionnaire;  Study site, age, gender, ethnicity, educational level attained, socio-economic status (SEI), and religion | | Socio-  economic index (SEI)  was  constructed from  commonly  available  household items  in a typical  Ugandan  households, has  previously been  used by this study group | To construct the SEI questions such as the following were used: *does your*  *household have*  *electricity?*  Response:  Yes/no | Administered to caregiver  Has previously  been used by this  study group. | (25) |
| **Caregiver factors** | | |  |  |  |  |
|  | Caregivers’ socio-demographics; age, gender, highest level of education, marital status and caregiver HIV status | | Questions that gathered information about caregivers’ socio-demographics | For example to assess caregivers’ highest level of education we asked the question:  *What is the highest level of education attained by caregiver?*  Responses:  1=No formal education  2= Primary level  3= Secondary level  4= University level  5= Other tertiary level | Administered to caregiver  Has previously  been used by this  study group. | (25) |
| To assess Caregivers’ psychological distress | Used the WHO Self-report Questionnaire (SRQ 20) | | 20-item questionnaire that assesses general psychological distress. | Has items such as: *Do you often have headaches?*  Response:  Yes/No | Administered to caregiver  A total score on the SRQ 20 is then calculated for each patient, with a clinically significant cut-off of ≥6 | Was culturally validated and used in Uganda (26, 27) |
| **Childhood’s psychosocial environment factors** | | |  |  |  |  |
| Felt HIV Stigma | Brief HIV Stigma Scale (B-HSS) | | 9-item questions on experiences,  feelings, and  opinions on HIV stigma; five items were used | Has items such: *I have been discriminated against at school/work because of being HIV positive*  Response:  Yes/no | Administered to adolescents  This 9-item scale is a psychometrically valid and reliable instrument that has both clinical and research applications | (28, 29) |
| Child-caregiver relationship | The Caregiver-Child Interaction Scale (CCIS) was used to assess for the child-caregiver relationship | | The Caregiver-Child Interaction is a 10-item, self-administered, communication scale, which can be completed by the parent/primary caregiver. It was adapted from the Child's Report of Parental Behaviour Inventory | The items were scored as follows;  1=never, 2=rarely, 3=sometimes, 4=often, 5=always. | In this study, four questions were used  This was administered to caregivers | (30) |
| Trauma | The Childhood Trauma Questionnaire-Short Form (CTQ-SF) | | 28-item  questionnaire on  traumatic events  in childhood; | Selected examples:  *Have you ever been beaten, hit, slapped?*  Response:  1= Yes  2= No | Two items were used in this study  This was administered to adolescents  Employed  for the second  time in Uganda by Kinyanda et al., 2016. | (31) |
| Food security | One item question | | Closed question | The item was; *In the last month,*  *did you or your*  *family have enough*  *food?*  Response:  Yes/no | This was administered to caregivers  It was Previously  used in the  HIV  situation of  Uganda by  Kinyanda et al., 2011. | (25) |
| Negative life  events | Two items from the modified European Parasuicide Interview Schedule | | Two questions on  adverse life  events  experienced in the  last one year | The two questions asked were:  *i) Did your parents separate?*  *ii) In the last year, was either of your parents seriously ill? e.g. got admitted.*  Responses:  yes/no | This was administered to adolescents  This tool has Previously  adapted to  the  Ugandan  sociocultural  context  and used in  HIV  research | (32) |
| **Child illness factors** | | |  |  |  |  |
| WHO clinical  stage for  HIV/AIDS | WHO Clinical  Staging criteria | |  | Respondents  classified as Stage I  to IV based on the  presence/absence a  combination of 17  HIV associated  clinical symptoms | This was administered to adolescents | (33) |
| CD4 counts | CD4 count taken  in the last 6  months | |  | Cells/ / µl of blood |  |  |
| Viral load | Viral load determined at assessment | |  | copies/ml |  |  |
| **Dependent Variables** | | |  |  |  |  |
| ADHD | DSM-5-referenced, behaviour rating scale, the Child and Adolescent Symptom Inventory-5 (CASI-5) | | ADHD presentations considered, namely ADHD inattentive, ADHD hyperactive, ADHD combined (i.e. both inattentive and hyperactive), any ADHD (i.e. either ADHD inattentive, ADHD hyperactive or ADHD combined).  CA-HIV was regarded as having ADHD combined if he or she reached the cut-off for both ADHD inattentive and ADHD hyperactive, while a CA-HIV was regarded as having any ADHD if they reached the cut-off for either ADHD inattentive or ADHD hyperactive or both | CA-HIV was considered to have the disorder if the number of symptoms in the category A which the caregiver rated as occurring “often” or “very often” reached a predetermined cut-off  Selected example:  *Does not pay close attention to details or makes careless mistakes*  Responses;  0= Never  1= Sometimes  2= Often  3= Very often | Eighteen items of the category A of the CASI-5 were used to assess for the different ADHD presentations. | (34) |
| **Negative clinical and behavioural outcomes** | | |  |  |  |  |
| Academic performance | | This section assessed the academic performance of the CA-HIV at school | Since academic performance in the Uganda education system is measured differently at the primary and secondary levels, we used 3 questions to develop a composite measure of poor academic performance. The 3 questions were:  *What was the academic performance of this child last term/semester?*  Response:  1=Poor 2=Fair  3=Good  4= Excellent  *What academic position did this child/adolescent hold in class last term/semester?*  Response:  Out of how many pupils/students  ………………. Number  *What aggregate points did this child/adolescent attain last term/semester?*  Aggregate points attained ………………….. | Poor academic performance at school which was determined as follows: in certain classes, performance is measured by a “points” aggregate, with lower aggregates denoting better performance. If the ratio of the points obtained to the best possible aggregate was greater than 12, then the CA-HIV was deemed to have performed poorly.  Alternatively if a points aggregate was not available, the CA-HIV was deemed to have performed poorly if his or her position in class was in the fourth quartile. If neither of these was available, then the performance in class was determined by the answer “poor” to the question “What was the academic performance of this child last term / semester?” | Asked of the caregiver | (35) |
| Experienced problems at school | | This section assessed for social functioning of the CA-HIV at school | We used 3 questions to develop the composite measure of ‘having experienced problems at school’ The 3 questions were:  *Did the child/adolescent suffer disciplinary measures (including suspension, dismissal) in the last term/semester?*    Response:  yes/no  *Did the child/adolescent stay away from school without permission in the last term/semester?*  Response:  yes/no  *Number of days missed at school in the last term*  Response:  Number ……………… | A CA-HIV was deemed to be positive for the composite measure, ‘having experienced problems at school’ if any of the following three conditions were met: i) A positive answer to the question *‘Did the CA-HIV suffer disciplinary measures (including suspension / dismissal) in the last term / semester?*’ ii) A positive answer to the question *‘Did the CA-HIV stay away from school without permission in the last term / semester?’* iii) The pupil was absent from school for 6 or more days in the last term / semester. | Asked of the caregiver | (35) |
| Risky sexual behaviour | | Involvement in sexual activity | Assessed sexual debut | Asked the question: *Have you ever had sex?*  Response:  yes/no | Only asked to adolescents | (25, 35) |
| Frequency of visits to the health unit | | One question item was used | Number of visits to the health unit in the past month | The question used to assess this was:  *How many times did you visit the health unit in the last month?*  Response:  Number of visits…………… | This was administered to adolescents only | (25, 35) |
| Frequency of hospital admissions | | One question item was used | Used the question:  *For how many days were you admitted to hospital in the last 6 months?*  Response:  Number of days………….  To create a derived variable. | Used responses to the question: *For how many days were you admitted to hospital in the last 6 months?* to create a derived variable of ‘whether or not the CA-HIV has been admitted to hospital in the last month. | This administered to adolescents | (25, 35) |
| Poor adherence to HIV treatment | | Used the 3 days recall test to assessed for non-adherence to HIV treatment | Used two questions to arrive at the composite measure of ‘being non-adherent to HIV treatment’.  The two questions were:  *For those on ARVs: How many days in the past 3 days have you missed taking ARVs?*  Response:  ……….  Number of days  *For those on Septrin/Dapsone: How many days in the past 3 days have you missed taking Septrin/Dapsone?*  Response:  ……….  Number of days | A CA-HIV had to meet the following conditions to be assessed as non-adherent to HIV treatment. If the participant was on ART, then failure to adhere was defined as having missed a dose of ART in previous three days. If the participant was not yet on ART, then failure to adhere was defined as having missed a dose of cotrimoxazole (CTX) prophylaxis in the previous three days. | This was administered to adolescents only | (25, 35) |
