# Supplementary material for: Prevalence and correlates for ADHD and relation with social and academic functioning among children and adolescents with HIV/AIDS in Uganda
Source: BMC Psychiatry. 2017 Sep 22;17:336. doi: 10.1186/s12888-017-1488-7 (PMC5610431; doi:10.1186/s12888-017-1488-7)
Supplement: Supplementary file 2 — Characteristics of study participants. (DOC 47 kb) [file 12888_2017_1488_MOESM2_ESM.doc]

| **Variable** | **Level** | **Total**  **(n=1,339)**  **n (%)** | **Children**  **(n=860)**  **n (%)** | **Adolescents (n=479)**  **n (%)** |
| --- | --- | --- | --- | --- |
| **Study Site** | Urban  Rural | 684 (51.1%)  655 (48.9%) | 424 (49.3%)  436 (50.7%) | 260 (54.3%)  219 (45.7%) |
| **Sex** | Male  Female  Missing | 638 (47.6%)  699 (52.2%)  2 (0.2%) | 413 (48.0%)  446 (51.9%)  1 (0.1%) | 225 (47.0%)  253 (52.8%)  1 (0.2%) |
| **Religion** | Christian  Muslim  Others/missing | 1058 (79.0%)  273 (20.4%)  8 (0.6%) | 676 (78.6%)  179 (20.8%)  5 (0.6%) | 382 (79.8%)  94 (19.6%)  3 (0.6%) |
| **Tribe** | Baganda  Non-Baganda  Missing | 967 (72.2%)  370 (27.6%)  2 (0.2%) | 629 (73.1%)  230 (26.7%)  1 (0.1%) | 338 (70.6%)  140 (29.2%)  1 (0.2%) |
| **Child lives with** | Both parent  Single parent  Grandparents  Others/missing | 354 (26.4%)  512 (38.2%)  258 (19.3%)  215 (16.1%) | 257 (29.9%)  340 (39.5%)  168 (19.5%)  95 (11.1%) | 97 (20.2%)  172 (35.9%)  90 (18.8%)  120 (25.1%) |
| **Orphanhood** | Single parent orphan  Double parent orphan  Non-orphan | 466 (34.8%)  152 (11.4%)  721 (53.8%) | 265 (30.8%)  57 (6.6%)  538 (62.6%) | 201 (42.0%)  95 (19.8%)  183 (38.2%) |
| **Highest level of education attained** | No formal  Pre-primary  Primary  Secondary  Missing | 29 (2.2%)  217 (16.2%)  954 (71.2%)  135 (10.1%)  4 (0.3%) | 12 (1.4%)  214 (24.9%)  629 (73.1%)  2 (0.2%)  3 (0.4%) | 17 (3.6%)  3 (0.6%)  325 (67.8%)  133 (27.8%)  1 (0.2%) |
| **Socio-economic index** | Mean(Std) | 4.43 (1.8) | 4.25 (1.8) | 4.76 (1.8) |
| **Socio-economic index (grouped)** | 0 – 2  3 – 4  5 – 6  7 – 9 | 194 (14.5%)  480 (35.8%)  480 (35.8%)  185 (13.8%) | 146 (17.0%)  330 (38.4%)  284 (33.0%)  100 (11.6%) | 48 (10.0%)  150 (31.3%)  196 (40.9%)  85 (17.8%) |
| **Current CD4 counts (cells / µl)** | <200  200-349  350-599  600-899  ≥900  missing | 63 (4.7%)  74 (5.5%)  229 (17.1%)  338 (25.2%)  621 (46.4%)  14 (1.0%) | 36 (4.2%)  22 (2.6%)  90 (10.5%)  175 (20.4%)  530 (61.6%)  7 (0.8%) | 27 (5.6%)  52 (10.9%)  139 (29.0%)  163 (34.0%)  91 (19.0%)  7 (1.5%) |
| **Child on ART?** | Yes  No | 1277 (95.4%)  62 (4.6%) | 824 (95.8%)  36 (4.2%) | 453 (94.6%)  26 (5.4%) |

**Additional file 2: Characteristics of study participants**
