# Supplementary material for: Prevalence and correlates for ADHD and relation with social and academic functioning among children and adolescents with HIV/AIDS in Uganda
Source: BMC Psychiatry. 2017 Sep 22;17:336. doi: 10.1186/s12888-017-1488-7 (PMC5610431; doi:10.1186/s12888-017-1488-7)
Supplement: Supplementary file 3 — Clinical correlates of ADHD. (DOCX 14 kb) [file 12888_2017_1488_MOESM3_ESM.docx]

**Additional file 3: Clinical correlates of ADHD-I, ADHD-HI, ADHD-C and any ADHD**

| **Child socio-demographic factors** | | **ADHD-Inattentive** | **ADHD-**  **Hyperactive-Impulsive** | **ADHD-**  **Combined** | **Any**  **ADHD** |
| --- | --- | --- | --- | --- | --- |
| **Factor** | **Level** | **aOR^1^**  **(95% CI; p-value)** | **aOR^1^**  **(95% CI; p-value)** | **aOR^1^**  **(95% CI; p-value)** | **aOR^1^**  **(95% CI; p-value)** |
| Site | Urban  Rural | 1 (Reference)    1.16 (0.63; 2.14)  ***P= 0.63*** | 1 (Reference)  0.99 (0.48; 2.04)  ***P=0.98*** | 1 (Reference)  1.67 (0.46; 6.12)  ***P=0.43*** | 1 (Reference)  1.09 (0.64; 1.84)  ***P=0.75*** |
| Sex of child | Male  Female | 1 (Reference)  0.68 (0.39; 1.20)  ***P=0.18*** | 1 (Reference)  0.67 (0.33; 1.39)  ***P=0.28*** | 1 (Reference)  0.68 (0.19; 2.48)  ***P=0.56*** | 1 (Reference)  0.67 (0.42; 1.09)  ***P=0.10*** |
| Age of child | Per 1 year increase | 1.14  (1.04; 1.25)  ***P=0.004*** | 0.96  (0.86; 1.08)  ***P=0.55*** | 1.09  (0.89; 1.34)  ***P=0.39*** | 1.09  (0.98; 1.14)  ***P=0.14*** |
| SES score | Per unit increase | 1.91  (1.17; 3.12)  ***P=0.009*** | - | - | 1.84  (1.21; 2.80)  ***P=0.004*** |
| **Caregiver factors** |  |  |  |  |  |
| Caregiver education level* | No formal / missing  Primary  Secondary  Higher | 1.79 (0.35; 9.27)  1 (Reference)  1.19 (0.35; 4.08)  4.08 (1.19; 13.96)  ***P=0.08*** | - | - | 2.36 (0.59; 9.51)  1 (Reference)  1.36 (0.45; 4.10)  4.01 (1.29; 12.48)  ***P=0.06*** |
| Caregiver SRQ-20 score | Per Unit increase | 1.11  (1.04; 1.18)  ***P=0.004*** | 1.11  (1.02; 1.20)  ***P=0.02*** | 1.16  (1.02; 1.33)  ***P=0.04*** | 1.11  (1.05; 1.17)  ***0.001*** |
| **Child psychosocial environment** |  |  |  |  |  |
| Orphan hood status | No parent alive  One parent alive  Both Parents alive | - | - | 11.03 (1.18; 103.34)  1 (Reference)  3.21 (0.37; 28.03) ***P=0.05*** | - |
| Quality of child-caregiver relationship | Per unit increase | 1.34  (1.22; 1.48)  ***P<0.0001*** | 1.20  (1.05; 1.37)  ***P=0.009*** | - | 1.32  (1.21; 1.44)  ***P<0.0001*** |
| Physical abuse (ever been beaten)* | Yes  No | - | 5.0  1 (Reference)  (1.25-20.83)  ***P=0.02*** | - | - |
| **Child illness factors** |  |  |  |  |  |
| Current CD4 count | Per 100 cell/µl increase | 0.88  (0.76; 1.01)  ***P=0.06*** | - | - | - |
| Worst WHO stage of child | I  II  III/IV | - | 7.35 (1.22; 44.25)  1 (Reference)  1.94 (0.42; 9.03)  ***P=0.11*** | - | - |

Note: *Assessed only among adolescents. aOR^1^ = adjusted Odds Ratio; adjusted for study site, age and sex
